# Parthenolide induces MITF-M downregulation and senescence in patient-derived MITF-M^high^ melanoma cell populations

**DOI:** 10.18632/oncotarget.7030

**Published:** 2016-01-27

**Authors:** Mariusz L. Hartman, Beata Talar, Malgorzata Sztiller-Sikorska, Dariusz Nejc, Malgorzata Czyz

**Affiliations:** ^1^ Department of Molecular Biology of Cancer, Medical University of Lodz, Lodz, Poland; ^2^ Department of Surgical Oncology, Medical University of Lodz, Lodz, Poland

**Keywords:** melanoma, MITF, NF-κB, parthenolide, cellular senescence

## Abstract

The activity of the M isoform of microphthalmia-associated transcription factor (MITF-M) has been attributed to regulation of differentiation, proliferation, survival and senescence of melanoma cells. MITF expression was shown to be antagonized by the activation of transcription factor NF-κB. Parthenolide, an inhibitor of NF-κB, has not been yet reported to affect MITF-M expression. Our results obtained in patient-derived melanoma cell populations indicate that parthenolide efficiently decreases the MITF-M level. This is neither dependent on p65/NF-κB signaling nor RAF/MEK/ERK pathway activity as inhibition of MEK by GSK1120212 (trametinib) and induction of ERK1/2 activity by parthenolide itself do not interfere with parthenolide-triggered depletion of MITF-M in both wild-type BRAF and BRAF^V600E^ melanoma populations. Parthenolide activity is not prevented by inhibitors of caspases, proteasomal and lysosomal pathways. As parthenolide reduces *MITF-M* transcript level and HDAC1 protein level, parthenolide-activated depletion of MITF-M protein may be considered as a result of transcriptional regulation, however, the influence of parthenolide on other elements of a dynamic control over MITF-M cannot be ruled out. Parthenolide induces diverse effects in melanoma cells, from death to senescence. The mode of the response to parthenolide is bound to the molecular characteristics of melanoma cells, particularly to the basal MITF-M expression level but other cell-autonomous differences such as NF-κB activity and MCL-1 level might also contribute. Our data suggest that parthenolide can be developed as a drug used in combination therapy against melanoma when simultaneous inhibition of MITF-M, NF-κB and HDAC1 is needed.

## INTRODUCTION

Melanoma is the most deadly form of skin cancer. Although immunotherapies and selective inhibitors of the BRAF/MEK/ERK pathway have improved outcomes for patients with advanced melanoma, the majority of melanomas either are intrinsically resistant or develop resistance after initial response [1–3]. This provides a strong rationale for combination therapies [2, 4]. To improve the current understanding of melanoma cell response to therapies, diverse studies have been initiated to analyze the genetic landscape determining an effective clinical response [2, 5, 6]. Novel therapies are in development and MITF is listed among attractive therapeutic oncotargets [3, 7]. MITF-M is a melanocyte-specific master regulator that has a critical role in the pathogenesis of melanoma and hyperpigmented disorders [8–12]. MITF-M regulation is complex and the exact mechanisms that determine MITF expression and activity remain incompletely understood [12–14]. MITF's contribution to phenotypic heterogeneity and plasticity of melanoma is considered as an important part of reduced sensitivity to the BRAF/MEK/ERK pathway inhibition [2, 7, 15].

Parthenolide (PN), a sesquiterpene lactone isolated from the herbal medicine feverfew (*Tanacetum parthenium*) inhibits proliferation and induces cell death in various cancers [16], remaining normal cells unaffected [17, 18]. Interestingly, PN shows activity against cancer stem-like cells [16, 18–21], and acts as a chemopreventive agent in a UVB-induced skin cancer model [22]. The major activity of PN is to inactivate specific proteins by forming a covalent bond between α-methylene-γ-lactone moiety of PN and sulfhydryl groups within proteins [23], and to increase intracellular concentrations of reactive oxygen species (ROS) [17, 24]. PN was first identified as nuclear factor κB (NF-κB) inhibitor [23] that can directly inhibit the p65 subunit [25], and it is still used as NF-κB inhibitor in a variety of studies [26, 27]. PN can also interfere with AP-1 [28] and STAT signaling [29] and induces activation of c-Jun *N*-terminal kinase (JNK) [30]. PN enhances p53 activity by promoting the ubiquitination of mouse double minute 2 homolog (MDM2) [31] and depletes histone deacetylase 1 (HDAC1) protein [32]. We and others have already shown diverse activities of PN in melanoma cells. In melanoma cell lines, A375, 1205Lu and WM793, PN inhibits proliferation and adhesion, induces cell cycle arrest, apoptosis and reduces constitutive and induced NF-κB activity [33, 34]. In SK-MEL-28 cells, PN rapidly induces ROS, and activates extracellular signal-regulated kinase 1/2 (ERK1/2) and NADPH oxidase [35]. In patient-derived melanospheres, it reduces the frequency of ATP-binding cassette, sub-family B, member 5 (ABCB5)-positive cells and clonogenic capacity [19]. The influence of PN on MITF-M has not been yet studied.

MITF-M expression in melanocytes and melanoma cells was shown to be suppressed by multiple inhibitors of HDAC1 [36]. As PN can inhibit HDAC1 in breast and colon carcinoma cells [32], we hypothesized that MITF-M level might be also diminished by PN in melanoma cells. However, it was also possible that PN could elevate the MITF level because it is an inhibitor of NF-κB and the gene expression reciprocity between NF-κB and MITF was reported in melanoma cell lines as modulating intrinsic sensitivity of melanomas to inhibitors of the BRAF/MEK/ERK pathway [37]. Therefore, we sought to clarify the influence of PN on MITF in NF-κB^low^ and NF-κB^high^ populations. To limit off-target effects, we did not modify cells genetically but instead we have chosen patient-derived melanoma populations with originally distinct molecular characteristics: (i) BRAF^WT^, MITF-M^high^/NF-κB^low^, (ii) BRAF^V600E^, MITF-M^high^/NF-κB^high^, (iii) BRAF^V600E^, MITF-M^low^/NF-κB^high^.

## RESULTS

### Patient-derived melanoma populations exert different doubling rate and basal expression of MITF/MITF-M and MITF-dependent genes

**Figure 1:**
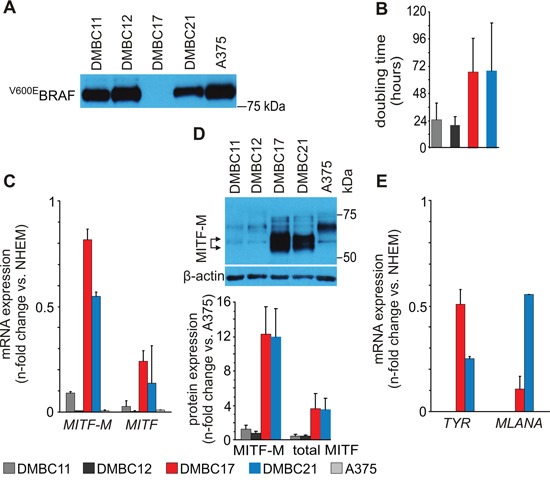
Molecular characteristics of patient-derived melanoma populations used in the study **A.** Western blot analysis of BRAF mutation status in patient-derived melanoma cell populations (DMBCs). An antibody recognizing BRAF^V600E^ but not wild type BRAF was employed. A375, a melanoma cell line harboring BRAF^V600E^ mutation was used as a positive control. **B.** Doubling time of melanoma cell populations assessed as metabolic activity of acid phosphatase. (n = 3) **C.** qRT-PCR analysis comparing basal levels of *MITF-M* and *MITF*. **D.** Western blot analysis comparing basal levels of MITF. A doublet of M isoform is indicated by arrows (top). MITF (total) and MITF-M protein levels were quantified relatively to their levels in A375 cells (bottom) (n = 3). **E.** qRT-PCR analysis comparing basal transcript levels of tyrosinase (*TYR*) and *MLANA*. Relative mRNA quantity of *MITF* and *MITF-M* (panel C), *TYR* and *MLANA* (panel E) is represented after normalization to *RPS17* and the level in melanocytes (NHEM). As in DMBC11 and DMBC12 cells the expression of *TYR* and *MLANA* was several hundred fold lower than in NHEM, it is displayed as zero. DMBC, patient-derived melanoma populations obtained in Department of Molecular Biology of Cancer.

Four patient-derived melanoma populations were grown in stem cell medium (SCM) as we have previously shown that this medium better preserves the parent tumor characteristics than serum-containing medium [38, 39, 40]. Three of them, DMBC11, DMBC12 and DMBC21 harbored mutation in BRAF (BRAF^V600E^), the most highly recurrent genetic aberration in melanoma [41] (Figure 1A). DMBC17 and DMBC21 were slow-cycling populations and their doubling time was above 60 hours, whereas doubling time of DMBC11 and DMBC12 populations was about 20-24 hours (Figure 1B).

**Figure 2:**
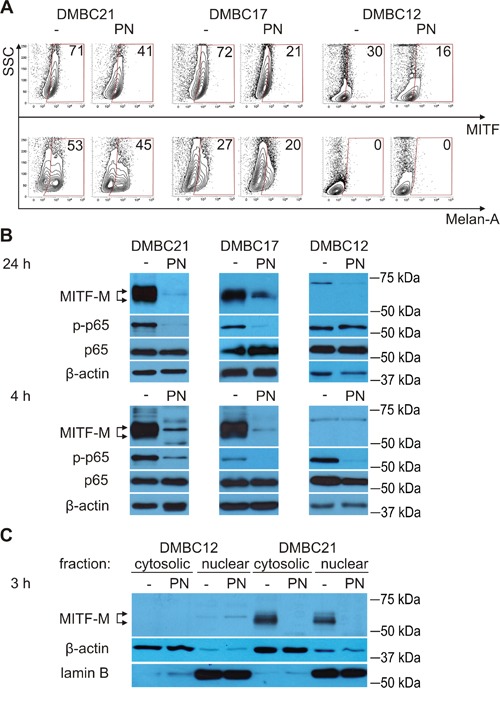
PN reduces percentages of MITF-positive cells and diminishes levels of MITF-M protein and phosphorylated p65/NF-κB **A.** Percentages of MITF (total)-positive and Melan-A-positive cells after treatment with 20 μM PN for 4 hours were assessed by flow cytometry. The numbers are representative percentages of MITF- or Melan-A-positive cells. **B.** Melanoma cells were treated with 10 μM PN for 24 hours or with 20 μM PN for 4 hours, and changes in the levels of MITF-M and phosphorylated p65 (p-p65) in whole-cell lysates were determined by Western blotting. **C.** MITF-M levels in cytosolic and nuclear fractions were analyzed by Western blotting after 3 hours of incubation with 20 μM PN. Equal loading was confirmed by β-actin (whole-cell lysates and cytosolic fraction) and lamin B (nuclear fraction). Representative results are shown.

*MITF-M* transcript was present in slow-cycling populations DMBC17 and DMBC21 at the level similar to that in melanocytes (NHEM), whereas *MITF-M* expression in DMBC11 and DMBC12 populations showing a high proliferation rate was very low as in A375 cells (Figure 1C). The most substantial difference between tested populations was observed in the basal level of MITF-M protein, which migrates as a doublet and it has lower molecular weight than other non-melanocyte-specific isoforms (Figure 1D). Concerning MITF-M activity, MITF-M-dependent pigmentation-related genes, *TYR* and *MLANA*, were expressed in DMBC17 and DMBC21 populations at similar levels as in melanocytes (NHEM) (Figure 1E). This indicates that DMBC17 and DMBC21 cell populations were highly heterogeneous and besides cycling cells they contained a large fraction of differentiated melanoma cells, which is further confirmed in Figure 2A. In DMBC11 and DMBC12 cells the expression of these genes was several hundred fold lower than in NHEM (Figure 1E). Thus, we had four melanoma patient-derived populations with different characteristics: (1) DMBC17: BRAF^WT^, MITF-M^high^, slow-cycling, highly differentiated, (2) DMBC21: BRAF^V600E^, MITF-M^high^, slow-cycling, highly differentiated and (3,4) DMBC11 and DMBC12: BRAF^V600E^, MITF-M^low^, fast-cycling, less differentiated.

### PN reduces the percentage of melanoma cells expressing MITF at high level

First, the influence of PN on the percentage of MITF (total)-positive cells was assessed using flow cytometry. At this stage of the study it was not clear whether PN would increase or reduce the percentage of MITF^high^ melanoma cells. After 20 hours, PN at 10 μM and 20 μM markedly reduced the percentage of cells expressing MITF at high level (not shown). More interestingly, this diminution was already visible after 4 hours (Figure 2A). Changes in the percentages of Melan-A-positive cells induced by PN were much lower (Figure 2A). Since MITF was substantially reduced by PN treatment, results for only one of MITF-M^low^ populations (DMBC12) were included.

### PN decreases MITF-M protein level in the nucleus and cytoplasm of melanoma cells

As the MITF antibody used in the flow cytometry detects all isoforms of MITF, the influence of PN on MITF-M isoform was subsequently assessed using Western blotting. MITF-M, a protein with a molecular weight between 50 and 65 kDa depending on its posttranslational modifications [12], was almost completely depleted by 20 μM PN in MITF-M^high^ populations after 24 hours, and similar effects were induced already after 4 hours (Figure 2B). PN eradicated MITF-M protein both in nucleus and cytoplasm as shown for MITF-M^high^ DMBC21 population (Figure 2C). We also noted that the inverse correlation between the level of MITF-M and phosphorylated p65 (NF-κB subunit) is not always true as we had DMBC21 cells that were MITF-M^high^/NF-κB^high^. Moreover, PN simultaneously reduced the level of both MITF-M and phosphorylated p65 (Figure 2B).

### PN-induced MITF-M depletion is proteasome-, lysosome- and caspase-independent

**Figure 3:**
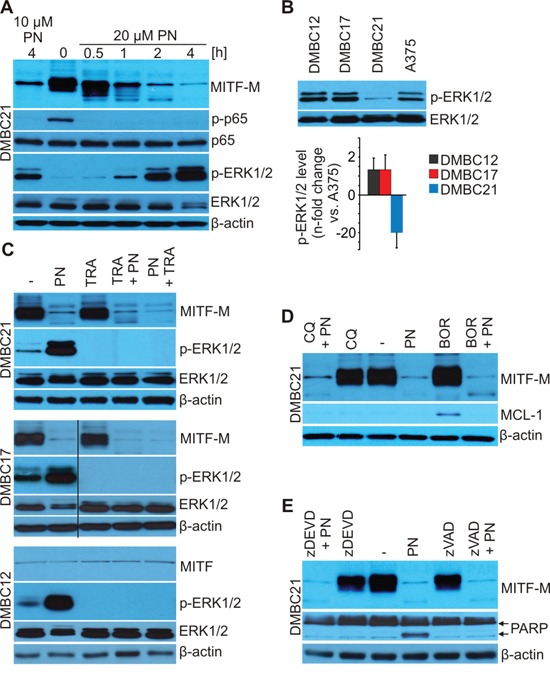
PN does not induce lysosomal, proteasomal and caspase-dependent degradation of MITF-M as shown by immunoblotting **A.** DMBC21 cells were treated with 10 and 20 μM PN for the indicated time and the levels of MITF-M, phosphorylated p65 (p-p65), p65, phosphorylated ERK1/2 (p-ERK1/2) and total ERK1/2 were assessed. **B.** Basal levels of p-ERK1/2 in DMBC cell populations shown by immunoblotting were quantified relatively to the level in A375 cells (n = 3). **C.** Cells were exposed to 20 μM PN and 0.5 μM trametinib (TRA) alone or in combination for 4 hours with 2 hours preincubation with either drug and protein levels were determined by Western blotting. **D.** and **E.** Cells were preincubated with bortezomib (0.1 μM BOR; 10 min), chloroquine (50 μM CQ; 10 min) or caspase inhibitors (50 μM zDEVD or 50 μM zVAD; 30 min) and 20 μM PN was added for additional 4 hours. MCL-1 was used as a control of BOR-triggered inhibition of proteasomal turnover, PARP as a control of caspase inhibition. Equal loading was confirmed by β-actin or ERK1/2. Representative results are shown.

The minimal time necessary for a substantial reduction of MITF-M protein level was determined (Figure 3A). MITF-M was rapidly reduced by 20 μM PN and already after 30 minutes first signs of protein depletion were detected, and after 4 hours almost no MITF-M was left. The phosphorylated p65 was completely eradicated already after 30 minutes further supporting the notion that MITF-M level does not inversely depend on NF-κB signaling (Figure 3A). Several mechanisms might be considered as responsible for MITF-M depletion. First, we investigated whether PN could induce ERK1/2 activity, which might contribute to the proteosomal degradation of MITF-M [42, 43]. When the membrane showing time-dependent depletion of MITF-M in DMBC21 cells was immunoblotted for phosphorylated ERK1/2, the activity of ERK1 and ERK2 was increased by PN also in a time-dependent manner (Figure 3A). When levels of phosphorylated ERK1/2 were assessed in untreated melanoma cells, it appeared that melanoma cell populations differed in basal levels of active ERK1/2 (Figure 3B), and ERK1/2^high^ populations, DMBC12 and DMBC17 expressed MITF-M at either very low or high levels, respectively. Therefore, it was rather unlikely that ERK1/2 activity was responsible for MITF-M level. To further confirm this conclusion, the activation of ERK1/2 was blocked by GSK1120212 (trametinib, TRA), an inhibitor of MEK. As expected, TRA at 0.5 μM used alone completely inhibited phosphorylation of ERK1/2 but did not affect MITF-M level (Figure 3C). In combination with PN, TRA did not prevent PN from action, and a full eradication of MITF-M was observed in MITF-M^high^ populations, DMBC17 and DMBC21 (Figure 3C). MITF-M depletion was observed even when ERK1/2 activity was first blocked with TRA and then PN was added. This clearly indicates that ERK1/2 was not involved in PN-induced MITF-M depletion.

MITF-M proteasomal degradation can be triggered by ERK1/2- [42] but also by glycogen synthase kinase 3 (GSK3)-dependent MITF-M phosphorylation [44]. To investigate contribution of proteasomal degradation to PN-induced MITF-M depletion, 0.1 μM bortezomib (BOR) was used but it did not prevent PN-induced downregulation of MITF-M (Figure 3D). To rule out the possibility that the lysosomal pathway is involved in the PN-induced loss of MITF-M, chloroquine (CQ), a lysosomal inhibitor was used. Chloroquine did not affect MITF-M level and did not interfere with PN-induced MITF-M depletion. As cleavage of MITF-M by caspases has been shown and interestingly, it generates fragment of MITF-M endowed with a proapoptotic activity [45], we sought to investigate whether caspases were responsible for PN-stimulated MITF-M degradation. Pretreatment with a pan-caspase inhibitor Z-VAD-fmk or caspase-3 inhibitor Z-DEVD-fmk did not affect PN-induced depletion of MITF-M (Figure 3E).

### PN reduces *MITF-M* transcript and HDAC1 protein level

**Figure 4:**
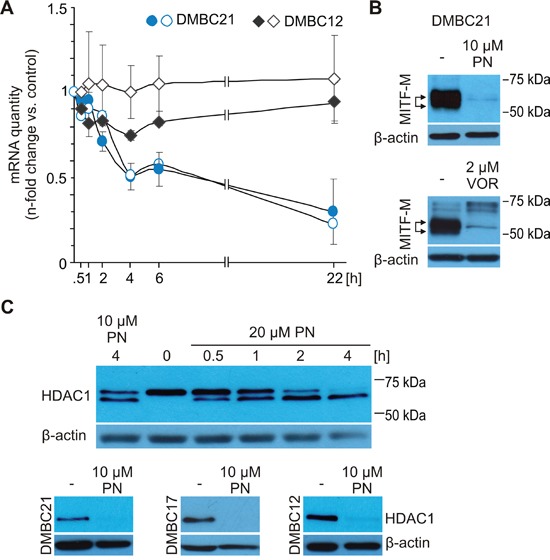
MITF level in melanoma cells might be reduced via inhibition of HDAC1 activity **A.** Expression of total *MITF* (closed symbols) and *MITF-M* (open symbols) was analyzed by qRT-PCR in DMBC21 and DMBC12 melanoma cell populations treated with 20 μM PN. n-fold change in mRNA quantity is represented after normalization to *RPS17* and the respective DMSO-treated control. **B.** Immunoblot analysis of lysates from DMBC21 cells treated with either 10 μM PN or 2 μM vorinostat (VOR) for 24 hours. **C.** DMBC21 cells were treated with 10 μM and 20 μM PN and harvested for Western blots at different time points to show changes in the HDAC1 level (top). HDAC1 level was assessed after 24 hours incubation with 10 μM PN (bottom). In Western blot experiments, equal loading was confirmed by β-actin. Representative results are shown.

As we excluded PN-induced degradation of MITF-M protein along any of known pathways, we next analyzed PN influence on MITF transcript level. qRT-PCR revealed that 20 μM PN substantially reduced mRNA levels of *MITF* and its *M* isoform in MITF-M^high^ populations DMBC21 (Figure 4A) and DMBC17 (not shown), whereas these transcripts expressed at low levels already in untreated DMBC12 cells (Figure 1C), remained unaffected by PN treatment (Figure 4A). Of note, the post-PN transcript level of MITF-M in DMBC21 population was still 3-fold higher than in DMBC12 population (not shown).

Previously, PN was shown to specifically inhibit HDAC1 in breast cancer cells [32]. Moreover, inhibition of HDAC1 was reported as the mechanism of MITF downregulation in melanoma [36]. Using vorinostat (VOR), an inhibitor of HDAC1 activity, we confirmed that MITF-M is down-regulated by HDAC1 inhibition also in MITF-M^high^ DMBC21 cell population (Figure 4B). The kinetics of PN-induced HDAC1 inhibition for DMBC21 cells is shown in Figure 4C, top. The faster migrating band showing the degradation product [46], was already present after 30 min with 20 μM PN (Figure 4C, top). HDAC1 cleavage was also observed in other three melanoma populations treated with 20 μM PN for 4 hours (not shown). The prolonged incubation with 10 μM PN caused complete disappearance of HDAC1 protein in all tested populations (Figure 4C, bottom).

### PN reduces proliferation, viability and clonogenic capacity of melanoma populations

**Figure 5:**
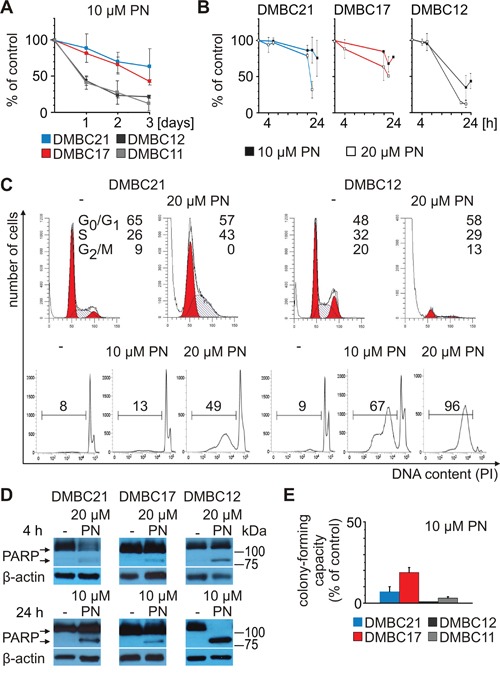
PN induces diverse cellular effects in different melanoma cell populations PN was used at the indicated concentrations. **A.** Changes in viable cell number were assessed after 1, 2, 3 days as acid phosphatase activity. **B.** Viability was estimated by flow cytometry after PI-staining. **C.** Changes in cell cycle were assessed by flow cytometry. Upper part shows histogram profiles generated with ModFit and the percentages of cells in the G_0_/G_1_, S and G_2_/M phases of cell cycle are indicated. The lower part shows the percentages of cells in subG_1_. **D.** Apoptosis is shown as PARP cleavage after 4 and 24 hours of incubation with PN. Equal loading was confirmed by β-actin. **E.** Percentage of clonogenic outgrowth of PN-treated versus untreated melanoma cells was assessed 3 weeks after stimulation.

PN inhibited cell proliferation and induced cell death displayed by an accumulation of cells in subG_1_ (Figure 5A, 5B and 5C). Induction of cell death was more efficient in DMBC12 population than in slow-cycling MITF-M^high^ DMBC21 population (Figure 5C). We have previously shown that PN induces apoptosis in melanoma cells [33, 34]. In the present study, poly(ADP-ribose)-polymerase (PARP) cleavage, a marker of apoptosis induction, was observed, and again it was more substantial in DMBC12 population than in DMBC17 and DMBC21 (Figure 5D). Exposure to PN for 4 hours was also long enough to markedly reduce a colony formation ability measured in soft agar after 3 weeks (Figure 5E).

### PN increases senescence in MITF-M^high^ melanoma cell populations

**Figure 6:**
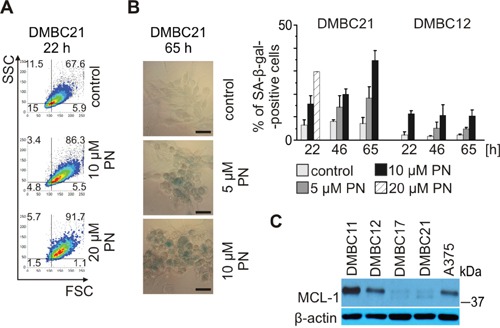
PN induces senescence in melanoma cells expressing MITF-M at high level PN was used at indicated concentrations. **A, B.** Senescence shown as (A) enlargement of cells (FSC) and increase in cell granularity (SSC) after 22 hours of treatment with PN and (B) changes in senescence-associated β-galactosidase (SA-β-gal) activity evaluated under the phase-contrast microscope (scale bar = 50 μm) and quantified (right). **C.** Basal levels of MCL-1 protein in melanoma cell populations analyzed by immunoblotting. Equal loading was confirmed by β-actin.

MITF-M depletion induces either senescence or apoptosis depending on the cellular background [12]. As PN was less efficient in triggering apoptosis in MITF-M^high^ populations, DMBC21 and DMBC17, we assessed whether it induced senescence. Indeed, 20 μM PN induced the hallmarks of senescence, such as (1) the enlargement of cells and increase in cell granularity already after 22 hours (Figure 6A), and (2) senescence-associated β-galactosidase (SA-β-gal) activity at acidic pH (Figure 6B) as shown for DMBC21 population. Similar results were obtained for DMBC17 population (not included). In contrast, not much stronger staining intensity was apparent in PN-treated MITF-M^low^ DMBC12 cells than in control cells (bars in Figure 6B). Thus, induction of senescence was accompanied by MITF-M depletion only in MITF-M^high^ melanoma cells. Of note, the basal activity of senescence-associated β-galactosidase was much lower in DMBC12 cells than in DMBC21 cells, and incubation with its substrate had to be prolonged from 4 hours (DMBC21) to 22 hours (DMBC12). Searching for other cell-autonomous differences which might account for lack of PN-induced senescence in DMBC12 population, we examined basal MCL-1 level in untreated melanoma cells. MCL-1 was already shown to reduce susceptibility of cancer cells to drug-induced senescence [47]. MITF-M^high^ populations, DMBC21 and DMBC17 had low levels of MCL-1, which might create the conditions suitable for PN-induced senescence, whereas high level of MCL-1 protein in DMBC11 and DMBC12 populations might restrict induction of senescence (Figure 6C). Of note, the expression of MCL-1 in DMBC21 cells was not deficient as this protein was accumulated in the presence of bortezomib (Figure 3E).

## DISCUSSION

MITF-M is a transcription factor specific for melanocytes and melanoma [8, 10, 12]. NF-κB is constitutively activated in many tumors [48, 49], and is involved in the cross talk with several transcription factors determining the cellular fate in response to stress [50]. The reduction of MITF expression has been reported as the result of the stimulation of melanoma cells with interleukin-1α or 1β suggesting that this process is NF-κB-dependent [51]. Moreover, the gene expression reciprocity between NF-κB and MITF in melanoma and a transition between phenotypes MITF-M^high^/NF-κB^low^ and MITF-M^low^/NF-κB^high^ as a part of melanoma plasticity in response to therapies targeting the BRAF/MEK/ERK pathway have been proposed [37]. The hypothesis of opposing regulatory networks operating through these two transcription factors prompted us to investigate the effect of PN, an inhibitor of NF-κB [16], on MITF-M expression in melanoma cells. Our results demonstrate, however, that PN very efficiently depletes MITF-M, both in the nucleus and in the cytoplasm. Furthermore, existence of the cell population that has MITF-M^high^/NF-κB^high^ phenotype (DMBC21) indicates that NF-κB not always antagonizes MITF expression in BRAF^V600E^-mutant melanomas. As (i) there was no tight inverse correlation between the level of MITF-M and phosphorylated p65 in untreated melanoma cells, and (ii) PN simultaneously reduced levels of both MITF-M and phosphorylated p65, a suppressive role of the NF-κB signaling on MITF-M protein level seems not plausible, at least for some melanomas. There are other examples of drugs simultaneously reducing MITF and NF-κB activities in melanoma cells [52, 53].

Senescence is recognized as an important process in the context of aging, wound healing and tumor suppression [54, 55]. In response to BRAF^V600E^ mutation, melanocytes form growth-arrested nevi that are characterized by expression of several markers of senescence [56]. In BRAF^V600E^ melanoma cells, vemurafenib induces features of senescence in addition to apoptosis [57]. In the present study, the induction of senescence by PN coincides with inhibition of MITF-M in MITF-M^high^ cells, whereas cell death was more efficiently induced in MITF-M^low^ cells. MITF suppression was already reported as accompanying a lineage-specific senescence program in melanoma cells [58]. It was also shown that cellular senescence is associated with activation of NF-κB, which mediates the proinvasive capacity of the senescence-associated secretory phenotype (SASP), and downregulation of NF-κB can reduce proinvasive properties of this secretome [59]. Thus, PN by simultaneous downregulation of MITF-M and NF-κB might induce senescence without triggering these deleterious effects, which might be beneficial for melanoma therapy. Chemotherapy-induced senescence was reported to be inhibited by MCL-1 [47]. Our results showing that senescence can be efficiently induced only in MCL-1^low^ melanoma cells support this finding.

PN was reported to specifically deplete HDAC1 protein in breast cancer cells without affecting other class I/II HDACs [32]. Our study has shown for the first time that PN can markedly decrease the HDAC1 level also in melanoma. MITF-M expression was already reported to be suppressed in melanocytes and melanoma cells by several HDAC inhibitors (HDACi), and this MITF depletion did not reduce melanocyte viability [36, 60]. Moreover, combined treatment with MAPK pathway and HDAC inhibitors suppressed MITF expression and melanoma resistance offering a novel clinical strategy to achieve more durable control of some BRAF^V600E^ melanomas [60]. Furthermore, a phase I clinical study of HDAC inhibitor panobinostat (LBH589) with ipilimumab with unresectable III/IV melanoma is currently recruiting participants (ClinicalTrials.gov; NCT02032810). Although the mechanisms of MITF downregulation by HDACi are poorly understood, they probably involve the interference with upstream transcriptional regulator(s) of MITF-M. Expression, stability and activity of MITF-M are dynamically controlled on multiple levels by several pathways, whose activities are determined by genetic background and the microenvironment-dependent physiological context [11, 12, 13]. In the present study, PN downregulates MITF-M expression level similarly to vorinostat, an HDAC1 inhibitor, and regardless of RAS/RAF/MEK/ERK pathway activity status. This effect is observed in both wild type BRAF and BRAF^V600E^, and inhibition of MEK by GSK1120212 (trametinib) and induction of ERK1/2 activity by PN itself do not interfere with PN-triggered depletion of MITF-M. Several studies indicate the close relationship between WNT/β-catenin signaling and MITF-M expression and stability [44, 52, 61]. We have reported previously that the microenvironment-driven suppression of the WNT/β-catenin pathway is accompanied by downregulation of MITF in MITF^high^ patient-derived melanoma cell populations [38]. Interestingly, the basal expression of LEF1, one of the *MITF-M* upstream regulators and effector of WNT signaling, was much higher in MITF-M^high^ than in MITF-M^low^ populations, and PN markedly reduced the mRNA level of LEF1 (not shown). As we excluded the influence of PN on proteasomal turnover or caspase-mediated MITF-M degradation, further studies are needed to delineate the mechanism(s) responsible for PN-driven MITF-M downregulation.

Our findings that PN efficiently reduces the levels of MITF-M and HDAC1 in melanoma cells, especially when combined with the previous results on its inhibitory effect on NF-κB activity, might have important implications for the clinics. PN exerts, however, low solubility and bioavailability as shown in a phase I dose escalation trial [62]. It was demonstrated that the solubility could be improved by using the fumarate salt of dimethyloamino parthenolide (DMAPT) [63, 64]. Another direction is to develop an efficient PN delivery system for cancer treatment and the work is ongoing [65]. But it should be also considered that this natural drug has been used for centuries as a bioactive component of feverfew (*Tanacetum parthenium*) to treat fever, migraine, rheumatoid arthritis and menstrual irregularities, and in this form is efficient and of low toxicity. Therefore, the present study strongly suggests that PN/feverfew could be considered as a part of prophylactic treatment or combined treatment when simultaneous inhibition of NF-κB, MITF-M and HDAC1 is needed. As targeting NF-κB enhances response to RAF inhibitors [66] and targeting MITF along with HDAC prevents cAMP/MITF-driven resistance to MAPK-pathway inhibitors [60], PN might be considered as a part of combined therapy for melanoma patients with BRAF^V600E^-driven melanomas.

**Figure 7:**
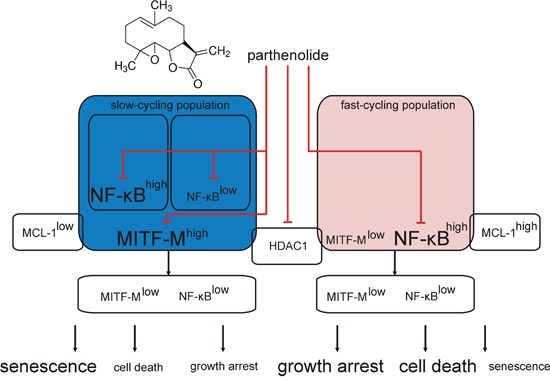
Schematic model of parthenolide (PN) activity in MITF-M^high^ and MITF-M^low^ patient-derived melanoma cell populations The present results clearly indicate that PN decreases MITF-M on the transcript and protein levels in MITF-M^high^ melanoma cell populations. Independently on the basal MITF-M expression level, PN inhibits the activity of p65/NF-κB and decreases HDAC1 level. Considering previous reports showing the MITF-M dependence on HDAC1 activity [36, 60] and PN capacity to suppress HDAC1 [32], we hypothesize that PN might influence the MITF-M level by inhibition of HDAC1. Diverse cellular effects of PN treatment are predefined by molecular characteristics of melanoma cell populations. In MITF-M^high^ melanoma cells either NF-κB^high^ or NF-κB^low^ (blue background) PN induces cellular senescence with minor cytotoxic effects, while in MITF-M^low^ populations (light red background) cell death is the dominant cellular outcome. Other cell-autonomous differences e.g., in the MCL-1 might also contribute. PN can be developed as a part of combination therapy against melanoma when simultaneous inhibition of MITF-M, NF-κB and HDAC1 is needed.

MITF protein levels vary between melanoma specimens [67], which is also reflected in the present study performed in patient-derived populations. Both high and low MITF-M expression levels have been linked with melanoma development. Alterations in the MITF-M level have been shown to induce changes in melanoma phenotype and function [13]. Inter- and intraindividual heterogeneity of melanomas results in the differential response to drugs [2, 68]. Our previous and present studies provide evidence that the character and intensity of cellular effects of PN can be diverse in different melanomas, and MITF-M level can be one of the determinants. The outcome of treatment with PN, cell death or senescence, might also depend on other molecules crucial for melanoma proliferation and survival such as MCL-1, and therefore melanoma tumors have to be molecularly characterized to select appropriate drugs for combined treatment. Taken together, the results of the present study provide data for a model (Figure 7) in which the possible pleiotropic effects of PN are narrowed by the molecular context exhibited in the present study as MITF-M and NF-κB levels. PN-induced inhibition of HDAC1 seems to be a common mechanism for all melanomas.

## MATERIALS AND METHODS

### Drugs and chemicals

Parthenolide (PN) was purchased from Biomol, Z-VAD-fmk from Promega (Madison, WI), Z-DEVD-fmk from BD Biosciences, chloroquine (CQ) from Sigma-Aldrich, bortezomib (BOR), vorinostat (VOR) and trametinib (TRA) from Selleck Chemicals LLC. Chemical structure of PN was prepared in ChemOffice 15.0.

### Tumor tissues

Melanoma specimens were obtained during surgical procedures. Permission was granted by Ethical Commission of Medical University of Lodz, and all patients consented to participate in the study.

### *In vitro* cell culture

Cells from tumor samples were isolated as described previously [69]. Briefly, tumor fragments were minced and incubated in Hank's balanced salt solution (Sigma-Aldrich) with 3 mmol/l calcium chloride, 1 mg/ml collagenase IV and 10 μg/ml DNase I at 37°C. Isolated cells were grown for one day in RPMI medium supplemented with 10% fetal bovine serum (FBS). Then, they were cultured in stem cell medium (SCM), consisting of DMEM/F12 low osmolality medium (Lonza), B-27 supplement (Gibco), growth factors: 10 ng/ml bFGF and 20 ng/ml EGF (BD Biosciences), insulin (10 μg/ml), heparin (1 ng/ml) and antibiotics (100 IU/ml penicillin, 100 μg/ml streptomycin). Medium was exchanged twice a week.

### Viability assay

Viability was assessed by propidium iodide staining according to standard procedures and analyzed using a FACSVerse flow cytometer (Becton Dickinson).

### Acid phosphatase activity (APA) assay

To assess relative changes in the viable cell number, the activity of acid phosphatase was measured colorimetrically [69]. Doubling time (DT) was calculated using the formula: DT = (*t − t_0_*)log2/(*logA − logA_0_*), in which *t* and *t_0_* are the times at which the cells were assessed and *A* and *A_0_* are the absorbance at times *t* and *t_0_*, respectively.

### Flow cytometry analysis of MITF- and Melan-A-positive cells

The following antibodies were used: anti-MITF (Abcam) along with APC-conjugated goat anti-rabbit (Santa Cruz Biotechnology) and anti-Melan-A (DAKO) along with FITC-conjugated goat anti-mouse (BD Pharmingen). Typically, 30,000 cells/sample were analyzed. Appropriate isotype controls were used. To exclude dead cells from the analysis, LIVE/DEAD® Fixable Violet Dead Cell Stain Kit (Invitrogen) was used. Acquisition was performed using FACSVerse flow cytometer (Becton Dickinson) and analyzed using BD Cell Quest software.

### Cell cycle analysis

Melanoma cells were treated with PN for 24 h. Cells were collected and fixed with 70% (w/v) ethanol at −20°C. After washing with PBS, cells were resuspended in PI Staining Buffer containing RNase (Becton Dickinson). Following incubation for 30 min at room temperature, cells were analysed using a FACSVerse flow cytometer. ModFit LT 3.0 software (Verify Software) was used to calculate the percentages in each cell cycle phase and FACSuite software (BD Biosciences) to calculate the percentages of cells in subG_1_.

### Preparation of cell lysates/fractions and immunoblotting

Preparation of cell lysates and immunodetection were described previously [70]. Briefly, melanoma cells were lysed in RIPA buffer (50 mmol/l Tris-HCl pH 8.0, 150 mmol/l NaCl, 1% Triton X-100, 0.5% sodium deoxycholate, 0.1% SDS) supplemented with freshly added protease and phosphatase inhibitors. To obtain cellular fractions, cells were homogenized in hypotonic buffer containing 10 mM HEPES, 1.5 mM MgCl_2_, 10 mM KCl, 0.2 mM PMSF and 0.5 mM DTT and cytosolic fraction was separated from nuclei that were lysed in RIPA buffer. Cell lysates and fractions were diluted in 2x Laemmli buffer and protein samples (15 μg each) were loaded on standard 7% SDS-polyacrylamide gel. The proteins were transferred onto an Immobilon-P PVDF membrane (Millipore). The membrane was incubated in a blocking solution: 5% nonfat milk in PBS-Tween 0.05% or 5% phosphoBLOCKER (Cell Biolabs) in PBS-Tween 0.05%. Primary antibodies detecting PARP, MCL-1, HDAC1, lamin B were from Santa Cruz Biotechnology, MITF, p-p65 (Ser536), p65, p-ERK1/2 (Thr202/Tyr204), ERK1/2 from Cell Signaling Technology. BRAF^V600E^ antibodies recognizing V600E-mutated, but not wild-type BRAF were from Biomol. Quantitative analysis of the results was performed by using NIH ImageJ software.

### RNA isolation and real-time PCR (qRT-PCR)

Total RNA isolation and qRT-PCR were described previously [71]. Briefly, RNA was isolated and purified using Total RNA Isolation kit (A&A Biotechnology). cDNA was subsequently synthesized by using random primers (Promega) and SuperScript II Reverse Transcriptase (Invitrogen). The amplification was performed by using KAPA SYBR FAST qPCR 2x Master Mix (Kapa Biosystems) and Rotor-Gene 3000 Real-Time DNA analysis system (Corbett Research). *RPS17* was used as a reference gene. The relative mRNA expression was calculated based on the expression ratio of the target gene *versus* reference gene, and correction of amplification efficiency of the individual transcripts was included as described by Pfaffl [72]. Sequences of primers used in qRT-PCR were shown elsewhere [38].

### Senescence assay

Senescence staining kit (Sigma-Aldrich) was used according to the manufacturer protocol. Cells were incubated with staining mixture for 4 hours (DMBC21) or 22 hours (DMBC12) and observed under a microscope (Olympus BX41; Olympus Optical). At least 200 cells were counted to calculate the frequency of senescence-associated β-galactosidase (SA-β-gal)-positive cells.

### Soft agar colony formation assay

Cells were incubated with PN for 4 hours and 1 × 10^3^ viable cells were transferred to plates containing agar as described previously [19]. Colonies were counted under the microscope 3 weeks later. PN-induced change in colony-forming capacity was expressed as % of control.
